# Supplementary material for: Hybrid Models and Biological Model Reduction with PyDSTool
Source: PLoS Comput Biol. 2012 Aug 9;8(8):e1002628. doi: 10.1371/journal.pcbi.1002628 (PMC3415397; doi:10.1371/journal.pcbi.1002628)
Supplement: Text S4 — Complete source code for the PyDSTool package (version 0.88.120504). Includes API documentation and help files linking to web pages. This file is identical to the current public release on Sourceforge.net. (ZIP) [file pcbi.1002628.s004.zip › PyDSTool/html/PyDSTool.Toolbox.phaseplane.nullcline_zone_leaf-class.html]

xml version="1.0" encoding="ascii"?


PyDSTool.Toolbox.phaseplane.nullcline\_zone\_leaf


| Home | Trees | Indices | Help | PyDSTool |
| --- | --- | --- | --- | --- |

Package PyDSTool ::
Package Toolbox ::
Module phaseplane ::
Class nullcline\_zone\_leaf

[hide private]

[frames] | no frames]

# Class nullcline\_zone\_leaf

source code

```
       object --+                
                |                
 MProject.feature --+            
                    |            
MProject.feature_leaf --+        
                        |        
 MProject.ql_feature_leaf --+    
                            |    
                    zone_leaf --+
                                |
                               nullcline_zone_leaf
```

Known Subclasses:
:   - fixedpoint\_zone
    - , inflection\_zone\_leaf
    - , max\_curvature\_zone\_leaf
    - , min\_curvature\_zone

---

Parameters to apply: pars: xtol, refine (integer, default 0) optional
pars: find\_exact\_center (unused)


|  |  |
| --- | --- |
| Instance Methods | [hide private] |

|  |  |
| --- | --- |
| \_prepare(self, nullc) | source code |

|  |  |
| --- | --- |
| \_set\_mutex\_zone\_by\_width(self, center\_ix, x\_center, y\_center, nullc)  Fix zone size by width or halfway to next center, if closer. | source code |

|  |  |
| --- | --- |
| \_grow\_zone\_by\_radius(self, center\_ix, x\_center, y\_center, nullc, property\_func) | source code |

|  |  |
| --- | --- |
| \_grow\_zone\_by\_width(self, center\_ix, x\_center, y\_center, nullc, property\_func)  property\_func must return +/- 1 or 0/1 as a function of x | source code |

**Inherited from `zone_leaf`**:
`evaluate`,
`finish`

**Inherited from `MProject.feature_leaf`**:
`__repr__`,
`__str__`,
`set_ref_traj`

**Inherited from `MProject.feature_leaf`** (private):
`_residual_info`

**Inherited from `MProject.feature`**:
`__call__`,
`__eq__`,
`__hash__`,
`__init__`,
`__ne__`,
`info`,
`postprocess_ref_traj`,
`prepare`,
`reset_metric`,
`validate`

**Inherited from `MProject.feature`** (private):
`_find_idx`

**Inherited from `object`**:
`__delattr__`,
`__getattribute__`,
`__new__`,
`__reduce__`,
`__reduce_ex__`,
`__setattr__`


|  |  |
| --- | --- |
| Properties | [hide private] |

**Inherited from `object`**:
`__class__`

| Home | Trees | Indices | Help | PyDSTool |
| --- | --- | --- | --- | --- |

|  |  |
| --- | --- |
| Generated by Epydoc 3.0.1 on Fri May 4 15:24:09 2012 | http://epydoc.sourceforge.net |
